# Assessing the Factors Leading to Missed Breast Cancer Diagnoses in Mammography Among Pakistani Women: A Prospective Cross-Sectional Study

**DOI:** 10.7759/cureus.71436

**Published:** 2024-10-14

**Authors:** Mariam Malik, Rana Bilal Idrees, Sadia Anwar, Farzana Kousar, Sharifa Sikandar, Muhammad Hamid Chaudhary

**Affiliations:** 1 Radiology, Atomic Energy Cancer Hospital, Nuclear Medicine, Oncology and Radiotherapy Institute, Islamabad, PAK; 2 Radiology, Institute of Nuclear Medicine and Oncology Lahore Cancer Hospital, Lahore, PAK; 3 Diagnostic Radiology, Institute of Nuclear Medicine and Oncology Lahore Cancer Hospital, Lahore, PAK; 4 Nuclear Medicine, Institute of Nuclear Medicine and Oncology Lahore Cancer Hospital, Lahore, PAK; 5 Cardiac Surgery, Chaudhry Pervaiz Elahi Institute of Cardiology, Multan, PAK

**Keywords:** breast cancer outcomes, breast lobular carcinoma, breast-radiology-mammography-ultrasound-mri-biopsyelastography, diagnostic mammography, metaplastic breast cancer

## Abstract

Objective

To determine the frequency of false-negative mammograms, and identify the factors contributing to missed breast cancer diagnoses in Pakistani women.

Materials and methods

This descriptive, prospective cross-sectional study was conducted at a tertiary care hospital from December 15, 2020, to December 10, 2023, including 150 women aged 30 to 60 who underwent bilateral mammography and concurrent breast ultrasound. The study analyzed the frequency and causes of false negatives, categorizing them into patient-related, tumor-related, technical-related, and provider-related factors. Stratification was performed based on age groups and Breast Imaging Reporting and Data System (BI-RADS) scores, and statistical significance was assessed using Chi-square tests.

Results

The study found a 5.1% frequency of false-negative mammograms. Lesion-related factors were seen in 59 (39.7%) patients; patient-related factors were seen in 40 (26.7%) patients; provider-related factors were seen in 29 (19.3%) patients; and technical-related factors were seen in 22 (26.7%) patients.

Conclusion

Dense breast tissue significantly contributes to missed breast cancer diagnoses in Pakistani women. While lesion-related, provider-related, and technical-related factors uniformly affect mammography outcomes, addressing patient-specific challenges - particularly in younger women with dense breasts - is crucial. The study suggests incorporating supplementary imaging modalities, like ultrasound, in routine screening for better detection, potentially informing national breast cancer screening guidelines in Pakistan.

## Introduction

Breast cancer is the leading cause of cancer-related deaths among women globally, with its incidence expected to reach 364,000 cases in the United States by 2040 [1]. Early detection is essential for reducing mortality, and mammography remains the gold standard for detecting malignant breast lesions, identifying approximately 75% of breast cancers at least a year before they become palpable [2]. This early detection is critical for improving treatment outcomes, quality of life, and prognosis. However, mammography's effectiveness varies significantly based on factors such as breast density, age, and screening quality. While substantial research has focused on mammography's effectiveness in Western populations, comprehensive studies on South Asian women - particularly in Pakistan - are lacking.

Mammography employs low-dose ionizing radiation and is considered safe, with benefits outweighing the risks. Screening mammography is recommended annually starting at the age of 40 for asymptomatic women [3] and earlier for those at higher risk [4], as advocated by the United States Preventive Services Task Force (USPSTF) and Society of Breast Imaging (SBI). Contrary to this, the British National Health Service (NHS) recommends screening for women between the ages of 50 and 70 years. Diagnostic mammography is used in symptomatic cases, helping determine the size and location of abnormalities [5]. The American College of Radiology's Breast Imaging Reporting and Data System (BI-RADS) standardizes imaging, interpretation, and reporting, with breast density categorized into four levels (A, B, C, D) - playing a crucial role in both mammogram interpretation and breast cancer risk [6,7]. Breast density is a mammographic finding that impacts detection rates, with sensitivity decreasing as density increases [8]. Women with dense breasts (Categories C and D) face a higher risk of breast cancer and are more likely to experience false-negative results due to the masking effect of dense tissue [9]. This is particularly relevant in Pakistani women, where Type C dense breast tissue is prevalent, highlighting the need for tailored screening protocols [10].

Unfortunately, early detection of breast cancer through mammography cannot be achieved if mammograms are not performed or reported correctly [11]. Studies indicate that 8-10% of breast lesions go undetected in mammograms. Combining ultrasound with mammography improves detection, particularly in dense breasts [12], as ultrasound can detect additional cancers that mammography alone might miss [13]. While not widely supported as a primary screening tool, ultrasound, when used alongside mammography, significantly improves diagnostic sensitivity [14,15]. Combining mammography with sonography yields sensitivity, specificity, positive predictive value, and negative predictive value of 90.4%, 82.4%, 95%, and 67%, respectively [16]. The use of sonography to differentiate benign and malignant masses has an accuracy of up to 88.9% [16]. Screening mammography alone has a sensitivity of 76% and a specificity ranging from 97% to 99% [17]. Other complementary techniques, like double reading, can further reduce false positives [18].

This study aims to determine the frequency of false-negative mammograms and the factors leading to missed breast lesions among Pakistani women. By analyzing the accuracy and limitations of mammography in this population and comparing it with additional imaging modalities like ultrasound, this research offers valuable insights for optimizing breast cancer screening strategies in resource-limited settings. The findings could influence national screening guidelines, advocating for personalized approaches based on age and breast density, ultimately improving breast cancer detection and outcomes in this region.

## Materials and methods

Our study was approved by the Institutional Review Board vide letter no: INMOL-53-(42). We conducted a descriptive, prospective cross-sectional study in the Radiology Department of a tertiary care hospital from December 15, 2020, to December 10, 2023, involving 150 patients out of 2,950 who underwent screening mammography. Data were collected using non-probability, consecutive sampling.

Inclusion criteria

Female patients aged 30 to 60 years who underwent bilateral screening mammography and were initially assigned a BI-RADS score of 0-3, with subsequent lesions detected on concomitant breast ultrasound, were included in the study. Patients younger than 40 years were advised to have screening mammograms based on their positive family history and BRCA1 or BRCA2 positive gene status. Patients initially assigned BI-RADS 4 or 5 were included if multifocal, multicentric, or contralateral carcinomas were missed on mammography but detected on ultrasound. All the patients included had subsequent biopsies of the lump performed for characterization.

Exclusion criteria

Patients without concomitant ultrasound or with only unilateral mammography were excluded from the study. Additionally, cases assigned BI-RADS 4 or 5 without detection of other multicentric, multifocal, or contralateral breast carcinoma were excluded. Our study was conducted on screening patients; hence, BI-RADS 6 was not included.

Data collection procedure

The procedures for mammography and breast ultrasound were explained, and informed consent was obtained. Mammography was performed using the Hologic Lorad Selenia (Hologic, Inc., Marlborough, MA, USA), capturing two standard views: craniocaudal and mediolateral oblique positions of both breasts. A technologist positioned the breast on the unit, applied compression, and suspended respiration during exposure to take X-ray images, with the entire procedure taking about 10 minutes. Digital mammogram images were obtained, with additional views (extended craniocaudal, spot compression, and magnification views) performed when necessary. A whole breast ultrasound was conducted using an Aloka ProSound 3500SX (Hitachi Medical Systems, Tokyo, Japan) or GE Logiq 500 (General Electric (GE) Healthcare, Chicago, IL, USA) with a high-frequency linear probe of 7.5 MHz. The mammograms were read by a senior radiologist with at least five years of experience and then re-read by a second senior radiologist. The same radiologists performed the breast ultrasound.

Causes of false negative mammography

The causes of missed carcinomas on the initial mammogram were analyzed and classified into four categories: patient-related, tumor-related, technical-related, and provider-related factors. The frequency of false-negative mammograms was recorded.

Tumor-Related Factors

Lesions missed in mammography due to tumor-related factors included subtle carcinomas (small <3 mm in size), signs of malignancy (areas of architectural distortion, small groups of amorphous or punctate microcalcifications, focal asymmetric densities, and dilated ducts), masked carcinomas (lesions hidden by overlying normal tissue, edema patterns, or those that may align along the normal tissue to mimic normal tissue), multifocal carcinomas (two or more cancers in the same quadrant or within 4 cm in one breast), multicentric carcinomas (two or more carcinomas in different quadrants or separated by 4 cm or more in one breast), and diffuse edema patterns. Edema of breast tissue is seen as skin thickening and stromal coarsening, and/or diffusely increased breast density with or without an associated mass and/or malignant-type microcalcifications. There may also be associated axillary lymphadenopathy.

Patient-Related Factors

Patient-related factors leading to missed lesions included dense breast tissue (high mammographic density appears as extensive white areas on an X-ray), uncooperative patients not allowing for proper positioning during mammography and motion artifacts.

Technical-Related Factors

Technical-related factors leading to missed lesions included malpositioned breasts. Findings on medio-lateral oblique views that indicate proper positioning include visualization of the pectoralis muscle to the level of the nipple, complete visualization of posterior breast tissue, breast tissue that is well compressed so that it is spread evenly, and the inframammary skin fold being visible. Findings on craniocaudal views that indicate proper positioning include the nipple being in profile and in the midline. Breasts should be evenly compressed in both views. Symmetrical images of both breasts are obtained.

Provider-Related Factors

Provider-related factors leading to missed lesions include poor perception and misinterpretation. The reporting radiologist who had missed the finding on the initial screening mammogram was given a performa to fill out, which included two reasons for not identifying the lesion correctly. Misinterpretation was defined as an abnormality with suspect features that was mistakenly reported as being definitely or at least probably benign. Poor perception was defined as missing a borderline abnormality mimicking the normal parenchymal appearances, such as a non-mass area of asymmetry or architectural distortion mimicking focal breast parenchymal densification. Discordant findings were resolved by longitudinal follow-ups and radiology board meetings, with discussions on equivocal cases to develop a consensus on imaging findings. 

Bias control

Technical bias was controlled by using the same mammography and ultrasound machines throughout the study. Observation bias was managed by having only two senior radiologists report the mammograms and perform the breast ultrasounds. Reporting was standardized by reviewing films in a mirror image pattern, using well-illuminated view boxes, and reviewing old films when available. BI-RADS scores were assigned to detected lesions, and selection bias was minimized through consecutive sampling.

Criteria for normal mammogram and breast ultrasound

A normal mammogram is categorized as BI-RADS 1, which demonstrates normal skin thickness, no definitive radiopaque or radiolucent lesions, preserved retromammary fat planes, and clear axilla or benign sub-centimeter lymph node morphology. On ultrasound, lesions that were oval/ellipsoid, wider than tall, with smooth margins, uniform homogeneity, or lateral shadowing with minimal posterior enhancement were considered benign or likely benign, corresponding to BI-RADS 2 or BI-RADS 3, respectively. Lesions with irregular margins, non-uniform homogeneity, absent lateral shadowing, microcalcification, microlobulation, intraductal extension, and increased echogenicity of surrounding fat were considered likely suspicious or highly suspicious for malignancy, corresponding to BI-RADS 4 or BI-RADS 5, respectively. However, there was a frequent overlap between benign and malignant lumps on both mammography and ultrasound; hence, histopathology correlation was necessary for definite characterization.

Data analysis procedure

Data were entered using IBM SPSS Statistics for Windows, Version 20 (Released 2011; IBM Corp., Armonk, NY, USA). Descriptive statistics were used to measure quantitative and qualitative variables. Factors leading to false-negative mammograms were included as qualitative variables, measured as frequencies and percentages. Patient age was included in quantitative variables and was measured as a mean with standard deviation. The percentage of patients according to BI-RADS scoring was also included in the quantitative variables. Effect modifiers, like age and BI-RADS scoring, were controlled by stratification, with post-stratification Chi-square tests applied. A p-value of ≤0.05 was considered significant.

## Results

The age range in this study was from 30 to 60 years, with a mean age of 46.85 ± 8.14 years. The majority of the patients, n = 69 (46.0%), were between 41 and 50 years of age, as shown in Table 1.

**Table 1:** Percentage of patients according to age distribution (n = 150).

Age (in years)	No. of patients	% age
30-40	33	22.0
41-50	69	46.0
51-60	48	32.0
Total	150	100.0

**Figure 1:**
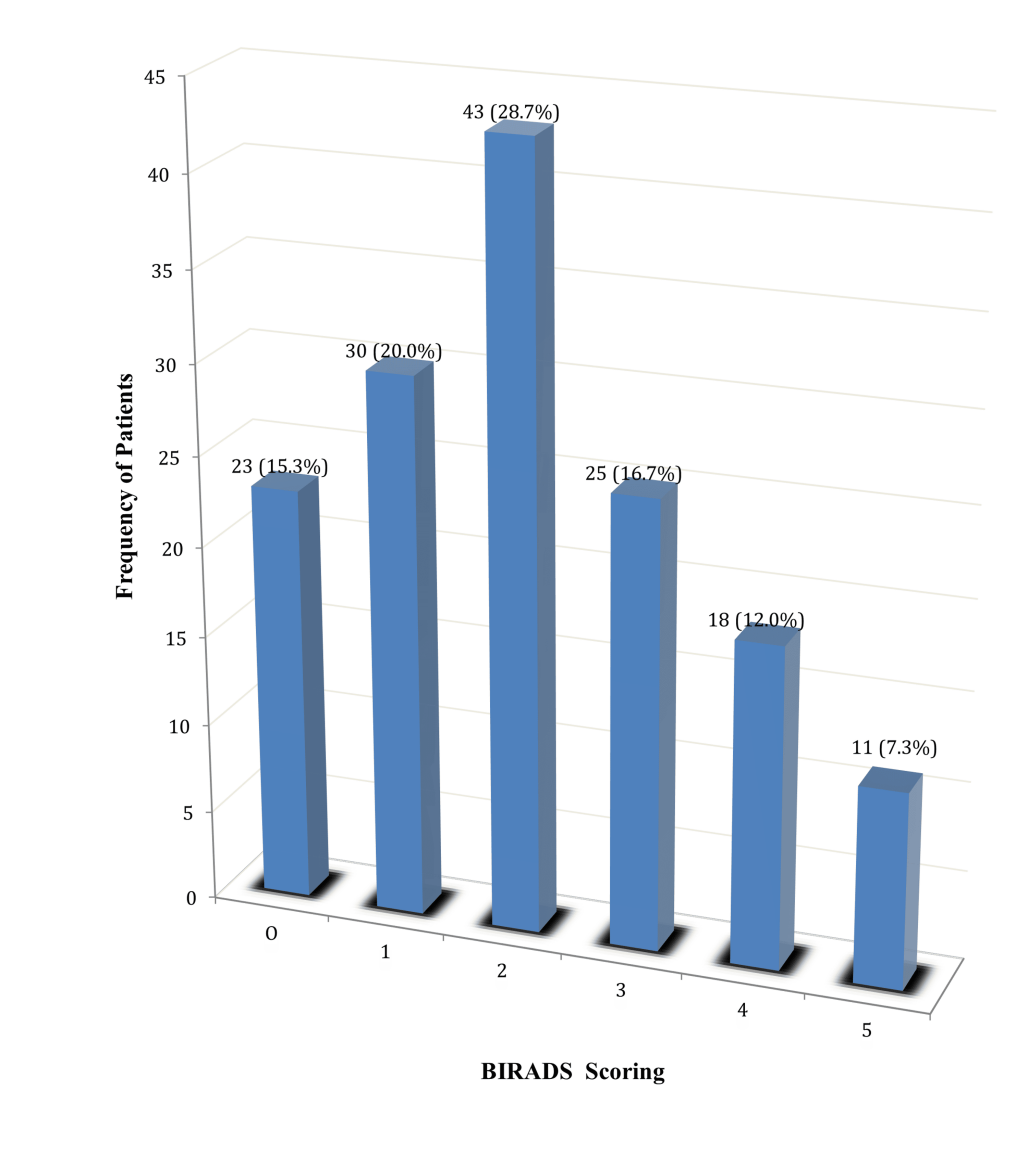
Percentage of patients according to BI-RADS scoring (n = 150). BI-RADS: Breast imaging reporting and data system

The percentage of patients according to BI-RADS scoring is shown in Figure 1.

We selected 150 patients with false-negative mammograms, which were selected after 2,950 consecutive mammograms, which showed the percentage of false-negative mammograms as 5.1%. Lesion-related factors were seen in 59 (39.3%) patients, patient-related factors were seen in 40 (26.7%) patients, provider-related factors were seen in 29 (19.3%) patients, and technical-related factors in 22 (14.7%) patients (Table 2).

**Table 2:** Factors leading to false-negative mammograms (n = 150)

Factors	No. of patients	% age
Lesion-related factors	59	39.3
Patient-related factors	40	26.7
Provider-related factors	29	19.3
Technical-related factors	22	14.7

**Figure 2:**
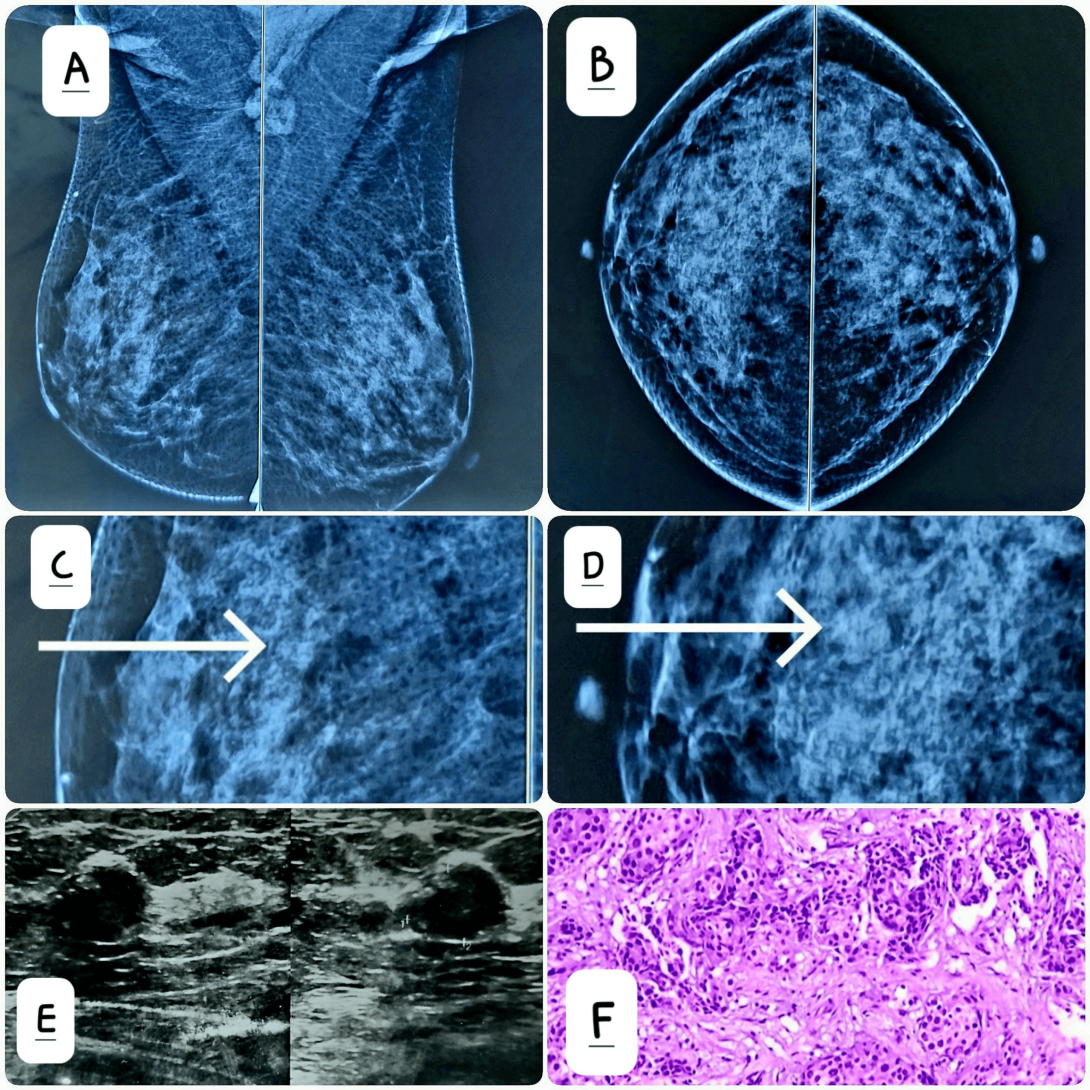
These images shows missed lesions in mammograms due to patient-related factors. Dense breast parenchyma (Type C) seen on mediolateral oblique (A) and craniocaudal (B) projections lowers the sensitivity of mammography. However, there is a faint, well-circumscribed radiodense opacity in the upper outer quadrant of the right breast (marked with a white arrow in images (C) and (D)). Correlative ultrasound (E) confirmed the presence of a well-circumscribed hypoechoic lesion. The finding was categorized as BI-RADS IV on imaging. Histopathology was performed (F), which confirmed invasive ductal carcinoma. BI-RADS: Breast imaging reporting and data system

Figures 2A-2F show missed lesions in mammograms due to patient-related factors (dense breast), which were subsequently detected on ultrasound.

The stratification of factors with respect to age groups is shown in Table 3.

**Table 3:** Stratification of factors with respect to age groups. Fisher’s exact test was used to calculate p-values. A p-value of less than 0.05 was considered significant.

Age groups	Lesion-related		Patient-related		Provider-related		Technical-related	
	Yes	No	Yes	No	Yes	No	Yes	No
30-40	13	20	11	22	03	30	06	27
41-50	22	47	24	45	12	57	11	58
51-60	24	24	05	43	11	37	08	40
p-value	0.143		0.008		0.271		0.968	

The stratification of factors with respect to BI-RADS scoring is shown in Table 4.

**Table 4:** Stratification of factors with respect to BI-RADS scoring. Fisher’s exact test was used to calculate p-values. A p-value of less than 0.05 was considered significant. BI-RADS: Breast imaging reporting and data system

BI-RADS scoring	Lesion-related		Patient-related		Provider-related		Technical-related	
	Yes	No	Yes	No	Yes	No	Yes	No
0	05	18	13	10	03	20	02	21
1	10	20	11	19	05	25	04	26
2	23	20	05	38	06	37	09	34
3	10	15	04	21	08	17	03	22
4	08	10	05	13	02	16	03	15
5	03	08	02	09	05	06	01	10
p-value	0.161		0.002		0.085		0.774	

## Discussion

Mammography is crucial for breast cancer detection, but it has limitations, especially concerning false negatives. These are difficult to quantify due to challenges in patient follow-up and the higher breast density often seen in younger women, which increases the likelihood of missed tumors [7]. Our study found that 5.1% of 2,950 consecutive mammograms resulted in missed diagnoses, highlighting areas for improvement in screening practices.

Lesion-related factors accounted for missed diagnoses in 39.7% of patients, with subtle carcinoma (23.3%) being the most common. These findings align with existing research, indicating the challenges of detecting small or masked tumors [19]. Patient-related factors were present in 40 (26.7%) patients. Dense breast tissue was a significant patient-related factor, consistent with prior studies, suggesting that mammography may be less effective in younger women with dense breasts [7,20,21]. This supports the need for supplementary imaging, like ultrasound or MRI, to improve detection in this population. Motion artifacts and patient non-cooperation were found to be less critical compared to breast density in influencing patient outcomes (Table 5).

**Table 5:** Number of patients with false negative mammograms due to lesions factors categorized according to age, breast density related and non-breast density related factors.

Age (years)	False-negative results		Total false negatives (out of 40)
	Breast density related (categories C and D parenchyma)	Non-breast density-related factors (patient movement and uncooperative patients)	
30-40	18	02	20
40-50	10	03	13
50-60	02	05	07

Provider-related factors contributed to missed diagnoses in 19.3% of cases, often due to misinterpretation (in 15 patients) or poor perception (in 14 patients), echoing findings from other studies [22]. Despite advancements like double reading and computer-aided diagnosis (CAD), interpretive errors remain, underscoring the need for ongoing training and development for radiologists. Technical-related factors, observed in 14.7% of cases, often involved improper technique, particularly in imaging tumors located peripherally or in the posterior breast area. Standardized positioning protocols and better training for technicians are crucial to addressing these issues [19].

The p-values reported in the study provide insights into the statistical significance of the various factors leading to missed lesions in mammography across different age groups. The p-value of 0.143 for lesion-related factors suggests that the association between lesion factors and age groups is not statistically significant. This indicates that, while lesion factors contribute to missed diagnoses, their impact does not vary significantly across different age groups. The similarity in the effect of lesion-related factors across age ranges might suggest that improvements in lesion-detection techniques would benefit all age groups similarly.

A p-value of 0.008 was seen for patient-related factors. This indicates a statistically significant association between patient-related factors (such as breast density) and age groups. This suggests that age influences how patient-related factors contribute to missed lesions. Specifically, the higher prevalence of patient-related factors leading to missed lesions in the 30-40 years age group implies that younger patients with dense breast tissue face greater diagnostic challenges. This finding underscores the need for tailored screening approaches for younger patients with dense breasts and suggests a need for age-specific screening strategies and further investigation into why certain factors disproportionately affect different age groups. Not only is breast density seen to contribute to more missed lesions, but it has also been observed in our study that older patients are more likely to have non-breast density-related factors affecting mammogram interpretation, including non-cooperative patients and patient movements. Therefore, screening could be tailored by counseling patients prior to the procedure, especially women older than 50 years, to avoid such factors that could influence image interpretation.

The p-value of 0.271 shows that provider-related factors (such as perception and interpretation errors) do not vary significantly across different age groups. While the breast density-related factor is the leading cause of perception and interpretation errors, when analyzed collectively, taking into account the non-breast density-related factors, the p-value does not turn out to be statistically significant among different age groups. This lack of statistical significance suggests that, while provider-related errors affect missed diagnoses, these errors do not disproportionately impact any particular age group. The consistent rate of provider-related errors across age groups may indicate that improvements in training and diagnostic support could benefit all patients equally.

A p-value of 0.968 indicates no significant variation in technical-related factors (such as mal-positioned breasts) across age groups. This suggests that technical issues impacting diagnostic accuracy are consistent, regardless of the patient’s age. Efforts to standardize and improve technical procedures should be universally applied, as technical-related factors do not seem to affect age groups differently.

The stratification results indicate the varying significance of different factors across BI-RADS scores. The p-value for lesion-related factors (0.161) suggests no statistically significant variation across BI-RADS categories, meaning lesion-related factors uniformly impact mammography results. A p-value of 0.002 for patient-related factors indicates a statistically significant association with BI-RADS scores, highlighting that patient-related variables, such as breast density, have a stronger influence on detection outcomes. The p-value for provider-related factors (0.085) is close to significant, suggesting that radiologist interpretation may differ slightly across BI-RADS categories, though not conclusively. Lastly, the p-value for technical-related factors (0.774) shows no significant variation across BI-RADS scores, indicating that technical issues, like breast positioning, affect mammography outcomes similarly across all categories.

Our study population, primarily aged 41-50, reflects the higher incidence of breast cancer in this age group. Analysis of missed lesions by age group reveals that patient factors significantly impact younger patients (30-40 years), while lesion factors are more prevalent in older groups (51-60 years). This suggests a need for age-specific screening strategies, including potentially lowering the screening age for high-risk women or integrating supplementary imaging techniques earlier. Statistical analysis of p-values further underscores the need for tailored screening approaches, especially for younger women with dense breast tissue, to reduce false negatives and improve early detection.

To the best of our knowledge, this study is the first to provide detailed insights into mammography performance in South Asian populations, specifically Pakistani women. The high prevalence of dense breast tissue in this cohort emphasizes the importance of incorporating supplementary imaging modalities into routine screening, particularly for women under 50. The study advocates for personalized screening protocols that consider age, breast density, and resource availability, potentially leading to earlier and more accurate diagnoses.

Our findings could influence healthcare policy in Pakistan, encouraging the adoption of more nuanced and effective breast cancer screening programs. Revising national guidelines to include recommendations for supplementary imaging in cases of high-density breast tissue could lead to earlier detection and better outcomes for women in this region.

Limitations of our study

Our study has a few limitations. The findings are based on a relatively small sample size and a specific cohort, which may limit the generalizability of the results to a broader population. Secondly, while technical errors account for a small percentage of missed cancers, they are still a factor. The study may not fully address the impact of these technical issues on overall diagnostic accuracy. Lastly, the findings are based on data from specific geographic regions or populations, which may limit their applicability to other settings with different breast cancer screening practices or demographic characteristics. Addressing these limitations in future research could provide a more comprehensive understanding of the factors affecting mammographic interpretation and cancer detection.

## Conclusions

This study underscores the complexity of mammographic screening and the multifactorial nature of missed lesions. Addressing tumor characteristics, enhancing patient-specific imaging, improving technical procedures, and reducing provider-related errors are essential steps to advance early breast cancer detection and reduce false negatives, which, in turn, would reduce breast cancer morbidity and mortality. Future research should focus on integrating advanced imaging techniques, improving standardization in mammographic practices, and enhancing radiologist training to address these challenges comprehensively.
